# Adverse Effects of Risperidone in Children with Autism Spectrum Disorders in a Naturalistic Clinical Setting at Siriraj Hospital, Thailand

**DOI:** 10.1155/2014/136158

**Published:** 2014-02-03

**Authors:** Vitharon Boon-yasidhi, Pantipa Jearnarongrit, Patnaree Tulayapichitchock, Jariya Tarugsa

**Affiliations:** Department of Pediatrics, Faculty of Medicine Siriraj Hospital, Mahidol University, 2 Prannok Road, Bangkok 10700, Thailand

## Abstract

A cross-sectional study was conducted to evaluate adverse effects associated with risperidone in 45 children with autism spectrum disorders (ASD), aged 2–15 years, who were treated at Siriraj Hospital, Thailand, between the years 2006 and 2007. Adverse effects were assessed by parent interview, using a semistructure questionnaire, and medical records review. The mean ± SD age of the children at starting risperidone was 8.15 ± 2.98 years. The mean ± SD of risperidone dose was 0.94 ± 0.74 mg/day and the mean ± SD duration of treatment was 36.8 ± 27.8 months. Adverse effects were reported in 39 children (86.7%). Common adverse effects included increased appetite, somnolence, and rhinorrhea and most of the adverse effects were tolerable. Tardive dyskinesia or other serious adverse events were not found in this study. The child's mean ± SD weight gain was 4.18 ± 2.82 kg/year, which exceeded developmentally expected norms. The results from this study suggest that risperidone treatment in children with ASD is associated with frequent mild and tolerable adverse effects. However, excessive weight gain could be found to be a concerning adverse effect and weight monitoring is warranted when risperidone is being prescribed.

## 1. Introduction

Autism spectrum disorders (ASD) are a group of neuropsychiatric conditions characterized by impaired social interaction and communication, along with restricted or stereotyped behaviors [1]. This group of disorders includes autistic disorder, Asperger's disorder, and pervasive developmental disorder, not otherwise specified. Besides the core symptoms, children with ASD may exhibit other behavioral symptoms such as irritability, hyperactivity, and aggression, which would further impair the child's developmental and social functioning [2]. Although educational and behavioral interventions are key components of treatment for children with ASD, medication management may be an additional treatment strategy for disturbing behavioral symptoms [3]. Among medication options, risperidone is the most commonly used for the treatment of serious behavioral problems in children with autism [4]. It has been also approved in Thailand for the treatment of behavioral problems associated with autistic disorder in children aged 5 years old and above.

A number of open label studies [5–8] and double-blind randomized placebo controlled trials (RCTs) [9, 10] have shown that risperidone was effective in treating behavioral symptoms and well tolerable in children with ASD. In two large 8-week RCTs by the Research Units on Pediatric Psychopharmacology (RUPP) Autism Network (*N* = 101, aged 5–17 years) [9] and Shea et al. (*N* = 55, aged 5–13 years) [10] risperidone was found to be effective in reducing irritability and associated with only mild to moderate adverse effects. Pool descriptive analysis from the two RCTs, reported in the risperidone prescribing information, indicated that the most common adverse effects of risperidone were somnolence (67%), increased appetite (49%), fatigue (42%), upper respiratory tract infection (34%), increased saliva (22%), constipation (21%), dry mouth (13%), tremor (12%), and dystonia (12%) [11]. In both studies, the average weight gain in risperidone group was 2.7 kg over the study period of 8 weeks, which was greater than that of the placebo group, and in excess of developmentally expected norms.

While existing data has demonstrated the safety of short-term treatment with risperidone in children with ASD, long-term safety data is limited. The objective of this study was to evaluate adverse effects of risperidone in children with ASD who received this medication for a long period in a naturalistic clinical setting at Siriraj Hospital, Thailand.

## 2. Material and Method

### 2.1. Study Design and Population

This was a cross-sectional study conducted during the years 2006-2007 at Siriraj Hospital, Mahidol University, which is one of teaching hospitals in Bangkok, Thailand. The Faculty of Medicine Siriraj Hospital Ethical Committee's approval (Si 005/2550) was obtained. The subjects were all children with ASD, aged 2–15 years, who received treatment in the child psychiatry clinic during the study period and were prescribed risperidone at some points of time. The ASD diagnoses were made according to DSM-IV diagnostic criteria. Children with cooccurring seizure disorders or other chronic medical conditions requiring medication management were excluded from the study. Consented parents were interviewed using a semistructured questionnaire asking if a child ever had any adverse effects of risperidone since the medication was started. The interviews took place during the child's regular clinic visits. The child's demographic and clinical data including weight, laboratory results, documentation of adverse effects, and abnormal movement examination were obtained from medical record review.

### 2.2. The Questionnaire

The questionnaire for assessing adverse effects of risperidone was developed by the investigators. It is comprised of questions asking parents to review whether the child has had experienced any of the listed 15 common adverse events or any other symptoms thought to be the adverse effects of risperidone at any point of time, since the medication was started. The list of common adverse events was complied based on the adverse effects of risperidone reported in the RTC studies [9, 10] and in the risperidone prescribing information [11]. The parents were also asked about the action taken for each reported adverse effect, that is, continuation, dose reduction, or discontinuation of the medication.

### 2.3. Statistical Analysis

Descriptive statistic was calculated for the frequencies, mean, and standard deviation of the demographic data and the reported adverse effects. The associations of each adverse effect and clinical variables were determined. Statistical analyses were performed using SPSS 16 statistical software package (SPSS Inc., Chicago, IL).

## 3. Results

During the study period, all of 46 children aged 2–15 years with ASD who received treatment in the child psychiatry clinic and ever received risperidone were screened; and 45 children were recruited. One child was excluded due to cooccurring seizure disorder being treated with antiepileptic medications. Forty children were diagnosed with autistic disorder and 5 children were diagnosed with PDD-NOS. The children's background characteristics were described in Table 1. More than three quarters of the children were male. The mean ± SD age at starting risperidone was 8.15 ± 2.98 years. The mean ± SD dose of risperidone was 0.94 ± 0.74 mg/day with the range of 0.25 to 4 mg/day. The mean ± SD duration of the treatment with risperidone was 36.8 ± 27.8 months, with the range of 1 month to 12 years. Indications for prescribing risperidone were hyperactivity and aggression in the majority of the children. Risperidone was the only medication prescribed in 68.9% and was coadministered with methylphenidate in 31.1% of the children. In the children who were taking both medications, the mean ± SD dose of methylphenidate was 26.5 ± 11.8 mg/day.

Adverse effects of risperidone were reported in 39 (86.7%) of the children. The three most common adverse effects were increased appetite (57.8%), somnolence (22.2%), and rhinorrhea (11.1%) (Table 2). These adverse effects were tolerable and no dose reduction or discontinuation of the medication was required in majority of the children. Risperidone was discontinued in 5 children, due to irritability in one child and due to symptoms improvement in 4 children. Medical record review revealed no occurrence of tardive or withdrawal dyskinesia, symptoms indicating hyperprolactinemia, or any other adverse events requiring laboratory investigation or therapeutic interventions. Complete data on weight was available in 42 children. Of those, the mean ± SD weight gain was 4.18 ± 2.82 kg/year.

Associations of each of the adverse effects with gender and age of the child, the dose of risperidone, the duration of the treatment, and the coadministration of methylphenidate were assessed. The results reveal no correlations between any of these pairs (data not shown).

## 4. Discussion

This study examined the occurrence of adverse effects of risperidone in children with ASD in a naturalistic university based clinical setting. We found that adverse effects of risperidone were reported in majority or more than 85% of the children. Serious adverse effects including tardive dyskinesia were not found in this study and medication was stopped in only one patient due to irritability. We, however, found excessive weight gain to be a concerning adverse effect in this population.

The adverse effects reported in this study were of mild intensity and tolerable similar to what were reported in the two placebo controlled risperidone trials [9, 10]. The dose of risperidone used in this study at the mean of 0.94 mg/day was slightly lower than that of the RUPP group (1.8 mg/day) [9] and Shea et al. (1.17 mg/day) [10]. The findings from the present study add to the evidence that, with a relatively low dose range, risperidone treatment in children with ASD is associated with frequent mild adverse effects.

In this study, we found no correlations between the occurrence of each of the adverse effects and the child's gender and age. Contrary to what we expected, adverse effects were not correlated with the dose of risperidone. This could be due to the fact that the dose of the medication used in this study was in a rather low and narrow range. We found no differences of the adverse effects in children who were on only risperidone and those who were on risperidone plus methylphenidate. This finding is similar to the study by Aman et al. that methylphenidate at regular therapeutic dosage does not have an influence in the occurrence of adverse effects of risperidone [12].

Although the chance of developing tardive dyskinesia with risperidone treatment is lower than that of typical antipsychotic medications, this condition remains one of the concerning serious adverse effects, particularly with long-term treatment. Although tardive dyskinesia was not found in any of the previous risperidone trials [9, 10], there was a case report of a child with autistic disorder who developed tardive dyskinesia after receiving risperidone for 23 months at a final dose of 3 mg/day [13]. In the present study, with the average treatment period of approximately 3 years, tardive dyskinesia was not found. This might be due to the fact that the dose of risperidone in this study was in a low range. Therefore, the risk of tardive dyskinesia associated with higher dose range cannot be excluded.

Similar to other studies [9, 10], we found weight gain to be concerning adverse effects associated with risperidone treatment. The average weight gain in our study was 4.18 kg/year, which exceeded developmentally expected norms. As excessive weight gain is associated with a number of medical complications, for example, diabetes mellitus, hyperlipidemia, and hypertension [14], this adverse effect should always be discussed with parents and regular weight monitoring is warranted when risperidone is being prescribed.

There are certain study limitations. First, the method of asking the parents to recall whether the child had ever experienced adverse effects could be subject to recall bias. Second, we did not perform laboratory investigation in every child; data on subclinical metabolic adverse effects of risperidone could not be drawn from this study. Third, there was no control group for comparison.

## 5. Conclusion

We have demonstrated that, in this naturalistic clinical setting, treatment with risperidone at the average of three-year duration in children with ASD was associated with frequent mild adverse effects. With a relatively low dose range used in this study, tardive dyskinesia was not found. However, we found that excessive weight gain was a concerning adverse effect. Physicians prescribing risperidone to children with ASD should discuss with the parents the possibility of developing adverse effects and monitor the child's clinical status closely.

## Figures and Tables

**Table 1:** Sample characteristics.

Characteristics	
Gender: male, *n* (%)	35 (77.8)
Mean (SD) age at starting risperidone, years	8.15 (2.98)
Mean (SD) dose of risperidone, mg/day	0.94 (0.74)
Mean (SD) duration of treatment, months	36.8 (27.8)
Indications for treatment with risperidone	
Hyperactivity, *n* (%)	30 (66.7)
Aggression, *n* (%)	5 (11.1)
Hyperactivity and aggression, *n* (%)	9 (20.0)
Irritability, *n* (%)	1 (2.2)
Other medications prescribed	
None, *n* (%)	31 (68.9)
Methylphenidate, *n* (%)	14 (31.1)

**Table 2:** Reported adverse effects of risperidone.

Adverse effects	Number	%
Increased appetite	26	57.8
Somnolence	10	22.2
Nasal congestion/rhinorrhea	5	11.1
Enuresis	4	8.9
Hypersalivation	3	6.7
Irritability/anxiety	3	6.7
Dry mouth	3	6.7
Abnormal movement	2	4.4
Dizziness	2	4.4
Insomnia	1	2.2
Constipation	1	2.2
Skin rash	1	2.2
Diarrhea	0	0
Nausea	0	0
Abdominal pain	0	0

## Abbreviation

ASD: Autism spectrum disorders.
